# *Citrobacter amalonaticus* Y19 for constitutive expression of carbon monoxide-dependent hydrogen-production machinery

**DOI:** 10.1186/s13068-017-0770-8

**Published:** 2017-03-28

**Authors:** Satish Kumar Ainala, Eunhee Seol, Jung Rae Kim, Sunghoon Park

**Affiliations:** School of Chemical and Biomolecular Engineering, Pusan National University, San 30, Jangeon-dong, Geumjeong-gu, Busan, 609-735 Republic of Korea

**Keywords:** *Citrobacter amalonaticus*, Water–gas-shift reaction, CODH, CO-Hyd, narG, gapA

## Abstract

**Background:**

*Citrobacter amalonaticus* Y19 is a good biocatalyst for production of hydrogen (H_2_) from oxidation of carbon monoxide (CO) via the so-called water–gas-shift reaction (WGSR). It has a high H_2_-production activity (23.83 mmol H_2_ g^−1^ cell h^−1^) from CO, and can grow well to a high density on various sugars. However, its H_2_-production activity is expressed only when CO is present as an inducer and in the absence of glucose.

**Results:**

In order to avoid dependency on CO and glucose, in the present study, the native CO-inducible promoters of WGSR operons (CO dehydrogenase, CODH, and CODH-dependent hydrogenase, CO-*hyd*) in Y19 were carefully analyzed and replaced with strong and constitutive promoters screened from Y19. One engineered strain (Y19-PR1), selected from three positive ones after screening ~10,000 colonies, showed a similar CO-dependent H_2_-production activity to that of wild-type Y19, without being affected by glucose and/or CO. Compared with wild-type Y19, transcription of the CODH operon in Y19-PR1 increased 1.5-fold, although that of the CO-*hyd* operon remained at a similar level. To enhance the activity of CO-Hyd in Y19-PR1, further modifications, including an increase in gene copy number and engineering of the 5′ untranslated region, were attempted, but without success.

**Conclusions:**

Convenient recombinant Y19-PR1 that expresses CO-dependent H_2_-production activity without being limited by CO and glucose was obtained.

**Electronic supplementary material:**

The online version of this article (doi:10.1186/s13068-017-0770-8) contains supplementary material, which is available to authorized users.

## Background

Carbon monoxide (CO), though toxic, is a valuable resource for biological H_2_ production via the so-called water–gas-shift reaction (WGSR) [1].

$$ {\text{CO }} + {\text{ H}}_{ 2} {\text{O }} \to {\text{ CO}}_{ 2} + {\text{ H}}_{ 2} ;\,\,{\text{DG }} = \, - 20{\text{ kcal mol}}^{ - 1}. $$

Although several microorganisms perform the WGSR, their use is restricted due to various challenges encountered in cell growth and biocatalytic activity. Most of these microorganisms require high temperatures and lavish nutrients (e.g., hyperthermophilic bacteria such as *Thermococcus onnurineus* and *Morella thermoacetica*) or sunlight (e.g., photosynthetic bacteria such as *Rhodospirillum rubrum*, *Rubrivivax gelatinosa,* and *Rhodopseudomonas palustris*) for growth and expression of WGSR enzymes [2–4]. Moreover, they usually grow only slowly and to a low cell density. Our laboratory strain, a chemotrophic Enterobacter, *Citrobacter amalonaticus* Y19, not only can perform WGSR but also grows rapidly to a high cell density on diverse and cheap carbon sources [5].

**Fig. 1:**
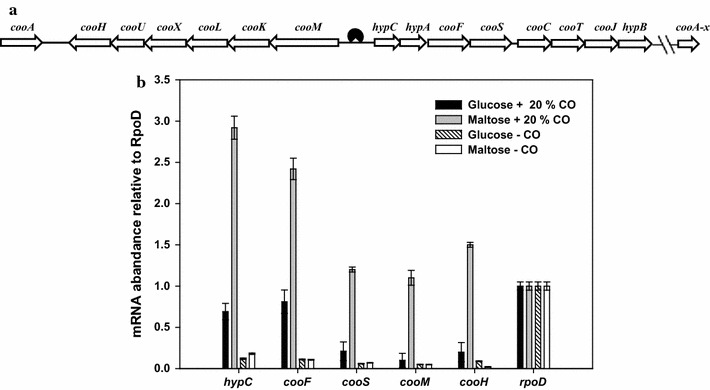
Gene organization of CO oxidation machinery (**a**) and carbon catabolite repression on expression of *coo* operon genes in *C. amalonaticus* Y19 (**b**). Binding of the transcriptional activator CooA in the intergenic regions of *cooM* and *hypC* is shown in (**a**). In **b**, the relative mRNA levels for various structural genes involved in CO-dependent H_2_ production during cell growth on glucose and maltose, respectively, are shown. Cells were grown both in the presence and absence of CO

Hydrogen production by WGSR usually is carried out by a two-stage process. In the first stage, cells are rapidly grown on a good carbon source with low or no H_2_ production, and in the second stage, H_2_ is produced with low or no cell growth. One challenge in the case of Y19 is that glucose, albeit the best carbon source for cell growth, does not induce CO-dependent H_2_-production activity, probably due to carbon catabolite repression (CCR) [6]. On the other hand, disaccharides such as sucrose and maltose efficiently promote H_2_-production activity but do not support fast growth of Y19. To deal with this problem, cells have been first cultured on glucose and then shifted to conditions under which WGSR activity is induced [7]. However, this growth-WGSR activity expression (two-step) strategy extends the culture period. Another challenge with Y19 is that, as with other microbes, its H_2_-production activity continuously declines in the production stage at which cell growth is almost stopped. Intermittent reactivation and/or maintenance of slow growth by addition of certain essential nutrients is necessary for prolonged H_2_ production. During the reactivation stage, *C. amalonaticus* Y19 strictly requires CO as an inducer of H_2_-production activity. The transcription of two-enzyme complexes for WGSR, namely carbon monoxide dehydrogenase (CODH) and carbon monoxide-dependent hydrogenase (CO-Hyd), which are encoded by two separate operons (Fig. 1), is positively regulated by the transcriptional activator protein CooA, and without CO, CooA cannot activate the transcription of the two-enzyme complexes [8]. CO is toxic; indeed, it interferes with the synthesis of many proteins, and its presence renders reactivation slow and very inefficient.

The purpose of this study was to engineer *C. amalonaticus* Y19 strains for their best WGSR use. Specifically, the use of glucose as the carbon source and the removal of CO dependency for CODH and CO-Hyd expression were targeted. To this end, the regulatory region of the operons for the two-enzyme complexes was carefully analyzed and replaced by several constitutive promoters screened from Y19. Furthermore, to improve CO-linked H_2_-production activity, overexpression of non-membranous CO-Hyd genes at the plasmid level and chromosomal 5′UTR modification of membraneous CO-*hyd* genes were also attempted. The newly developed recombinant *C. amalonaticus* Y19 could grow on glucose and express CO-dependent H_2_-production activity constitutively, based on which results it was considered to be a highly convenient biocatalyst for the WGSR.

## Methods

### Construction of recombinant strains

*Citrobacter amalonaticus* Y19 isolated from an anaerobic waste-water sludge digester was utilized as the parental strain for development of recombinant strains [7]. Table 1 lists the strains and plasmids used in this study. For the construction of the promoter-replaced strains and deletion mutants, in-frame tagged deletion/replacement was performed in the chromosomal DNA according to the methods described by Link et al. with modifications [9]. Approximately 500 bp homologous regions upstream (fragment A) and downstream (fragment B) were PCR-amplified. In the case of promoter replacement, about 300 bp intergenic regions of selected constitutive promoters were PCR-amplified and sandwiched between the upstream and downstream fragments by overlapping PCR in order to obtain a replacement fragment (A-P_nar_*–P_gap_-B). On the other hand, in the case of deletion mutant construction, overlap between fragments A and B was performed to obtain a deletion mutant AB. Next, these fragments were cloned into the BamH1 and Not1 restriction sites of the pKOV vector and confirmed by sequencing. The resultant plasmid was subjected to double recombination in order to replace the native *coo* promoters with P_nar_*-   and P_gap_-constitutive promoters. Briefly, after transformation with pKOV plasmid (carrying engineered P_nar_*- and P_gap_-constitutive promoters), the recombinant Y19 cells were plated onto Luria–Bertani (LB) medium containing chloramphenicol (25 μM) and incubated at 42 °C. The positive integrant strains were then cured of the plasmid using sucrose (*sacB*-dependent) as selection pressure. The sucrose-resistant and chloramphenicol-sensitive colonies were screened by boil PCR for the desired gene replacement. The promoter-replaced mutant was designated as Y19-PR1.

**Table 1:** Bacterial strains and plasmids used in this study

Strains and plasmids	Genotype and description	Source
Strains		
Y19 WT	*Citrobacter amalonaticus* Y19; Amp^r^	[7]
Y19-PR1	Y19 with chromosomal replacement of native *coo* promoters with P_gap_ and P_nar_*	This study
Y19-PR2	Y19-PR1 with UTR1(threefold) *cooM*	This study
Y19-PR3	Y19-PR1 with UTR1(6.5-fold) *cooM*	This study
Y19-PR1/pHyd-CO	Y19-PR1 harboring pHyd-CO plasmid	This study
Y19-PR2/pHyd-CO	Y19-PR2 harboring pHyd-CO plasmid	This study
Y19-PR3/pHyd-CO	Y19-PR3 harboring pHyd-CO plasmid	This study
*E. coli* DH5 alpha	Cloning host	KCCM
Plasmids		
pUCPK/P_gap_ or P_nar_*	ColE1 ori, green florescent protein under the control of P_gap_ or P_nar_*	[23]
pKOV/ΔP_coo_::P_gap–nar*_	pKOV-pSC101-*sacB*; Cm^r^	[24]
pDK7	P15A ori; Cm^r^	[8]
pHyd-CO	pDK7 p15A P_nar_* *cooKLXUH*	This study

To verify the strengths of the selected native promoters (P_gap_ and P_nar_*) in *C. amalonaticus* Y19, about 300 bp of intergenic regions were PCR-amplified and cloned under green fluorescent protein (GFP) as a fluorescent marker.

### Culture conditions

*Citrobacter amalonaticus* Y19 was cultivated in modified M9 medium fortified with potassium phosphate buffer (100 mM; pH 7.0). The medium contained the following constituents: 1.0 g/L MgSO_4_·7H_2_O, 1.0 g/L NaCl, 1.0 g/L NH_4_Cl, and 3.0 g/L yeast extract. Maltose and glucose were used as the sole carbon sources at 5 g/L, respectively, wherever indicated. l-Cysteine (1.0 mM), sodium selenate (2 μM), sodium molybdate (2 μM), NiCl_2_ (10 μM), and FeSO_4_ (25 μM) were added to the culture medium as essential micronutrients supportive of cell growth. All the experiments were performed in 165 mL serum bottles (working volume, 50 mL) at 30 °C. The bottles were sealed with a butyl rubber septum and aluminum cap before inoculating the seed culture. The bottles were flushed with argon (Ar) gas (99.9%) for 10 min to ensure O_2_ deprivation. Unless stated otherwise, the culture head space contained an Ar/CO (80:20, v/v) mixture.

### Measurement of whole-cell and crude-cell extract enzymatic activities

The enzymatic activities of the whole-cell or crude-cell lysates were examined as described previously [8]. Briefly, the whole-cell activities were measured with cells harvested during the late exponential growth phase, washed twice with MOPS buffer (pH 7.0) and then resuspended in the same buffer. The cell suspensions were placed in a 9.5 mL serum bottle at 0.6–0.8 OD_600_ and charged with an Ar/CO (80:20, v/v) gas mixture to initiate CO-dependent H_2_ evolution. The activities of CODH and the hydrogenases (uptake and evolving hydrogenases) were measured using crude-cell lysates. The lysates were prepared by harvesting cells during the late exponential growth phase, washed twice with 100 mM cold phosphate buffer, and resuspended in 50 mM Tris–HCl buffer (pH 7.3, buffer A) containing 2 mM dithiothreitol and 1 mM sodium dithionate. The cells were disrupted using a bead beater (Fastprep FP120, Obiogene Inc., USA) following the standard protocols. The enzymes were assayed at 30 °C in either 9.5 mL serum bottles or 4 mL cuvettes under anoxic conditions. The CODH activity was determined by methyl viologen-dependent CO oxidation, as described previously [8]. The reaction mixture, containing 15 mM of MV, 2 mM of SDT, and 1 mM of EDTA in 3[*N*-morpholino] propanesulfonic acid (100 mM MOPS; pH 7.0) buffer, was introduced into an anaerobic 10 mm quartz cuvette and bubbled with pure CO for 5 min, after which a sufficient amount of enzyme solution (0.6–0.8 mg mL^−1^) was added to initiate the reaction. The reduction of MV was monitored at 578 nm using a double-beam spectrophotometer (Lambda 20, Perkin Elmer, USA). For determination of the uptake hydrogenase activity, the oxidized form of MV was used as an electron acceptor, and the MV reduction was measured colorimetrically at 578 nm. The reaction mixture containing buffer A and the electron acceptor (MV) at 2 mM was equilibrated with 100% H_2_. The molar extinction coefficient ε_578_ for the reduced methyl viologen was 9.7 mM^−1^ cm^−1^. To assess the H_2_-formation activity, the reduced MV was used as an electron donor, and the MV-dependent H_2_ evolution was measured in the gas phase by gas chromatography.

### Fluorescence assay

For GFP-fluorescence measurements, 200 µL of culture was immediately chilled on ice and then measured (*λ*_ex_ = 485 nm; *λ*_em_ = 515 nm) on a Perkin Elmer/Wallac Victor 2 Multilabel Counter (1420-011). A non-GFP-producing *C. amalonaticus* Y19 culture was used as a blank for the fluorescence measurements.

### Real-time PCR

Wild-type and recombinant Y19 were cultivated in a modified M9 medium under anaerobic conditions at 30 °C and agitated at 250 rpm in an orbital incubator shaker. The cells were harvested during the stationary growth phase. Approximately 2 × 10^8^ cells were collected in vials containing two volumes of RNA protect reagent (Qiagen Inc., USA). The culture suspension was centrifuged at 10,000 rpm for 5 min. Pellets were applied for total RNA extraction using a total RNA isolation kit (Macherey–Nagel, Germany). Two micrograms of total RNA was used to synthesize the first-strand cDNA in a 20 µL reaction utilizing a SuperScript III first-strand synthesis system (Invitrogen, USA). Real-time PCR analysis was performed in a 20 µL reaction volume using the SYBR Green method with the StepOne Real-Time PCR system (Applied Biosystems, USA). Each 20 µL sample of the reaction mixture contained 300 ng of cDNA, 10 µL of 2× Power SYBR Green PCR Master Mix (Applied Biosystems, UK), 5 pmol of forward and reverse primers, and DEPC-treated water. The thermal cycling conditions were as follows: denaturation, 1 cycle of 95 °C for 30 s; amplification, 40 cycles of 95 °C for 15 s, 62 °C for 30 s, and 72 °C for 30 s. The PCR efficiencies of all the primers were determined experimentally and found to be suitable for reliable copy-number quantification. The relative quantification of the mRNA levels was calculated using the ΔΔCT method described previously [10, 11]. All the assays were performed in duplicate, and the reaction without a template was used as the negative control.

### Analytical methods

Bacterial growth was measured by spectrophotometry (Lamda 20, Perkin Elmer, USA) at 600 nm. The protein content was measured using the Bradford method with bovine serum albumin as the standard. The H_2_ and CO contents were quantified by gas chromatography (DS 6200; Doman Inst. Inc., Korea) equipped with a Thermal Conductivity Detector, utilizing stainless steel columns packed with Molecular Sieve 5A (for H_2_; Alltech Deerfield, IL, USA). The injector, column oven, and detector temperatures were 90, 80, and 120 °C, respectively. Argon was used as the carrier gas at a flow rate of 30 mL min^−1^. Organic acid and alcohol analyses were carried out by HPLC (1100 series Agilent Technologies Foster, CA, USA).

## Results and discussion

### Effect of glucose on transcription of CO-dependent H_2_-production enzymes

According to a previous study [6], the WGSR activity of Y19, measured in both whole-cells and broken-cell extract, was negligible when the cells were grown on glucose. To confirm the mechanism, the transcription of a few selected genes in the two CO-responsible operons, CODH and CO-*hyd*, were analyzed in the wild-type *C. amalonaticus* Y19 (Fig. 1). The cells were cultured on either glucose or maltose, in the presence and absence of CO. Even with CO, wild-type Y19 grown on glucose showed a significantly lower transcription for the CODH (*hypC*, *cooF*, *cooS*) and CO-*hyd* (*cooM*, *cooH*) genes compared with the cells grown on maltose. Without CO, expression of all *coo* operon genes was low and, furthermore, not much difference between the two carbon sources was noticed. The highest transcription was exhibited when the cells were grown on maltose with CO. These results indicate that expression of the CODH and CO-*hyd* operons is under dual control at the transcriptional level by CO and the carbon source (CCR). Interestingly, the fold difference between the two carbon sources in the presence of CO was only 3–10, much smaller than that by CO (~30, grown on maltose) or that found in the well-known *lac* operon (~100) [12].

Transcription of the CODH genes gradually decreased at locations farther from the promoter site, due possibly to the polarity effect. By contrast, two genes of the CO-*hyd* operon, *cooM*, and *cooH*, though distantly located in the operon, did not show such difference. We also noticed that the CODH genes, *hypC* and *cooF*, were highly transcribed compared with *cooM* and *cooH*.

### Analysis of CO-inducible promoter region

**Fig. 2:**
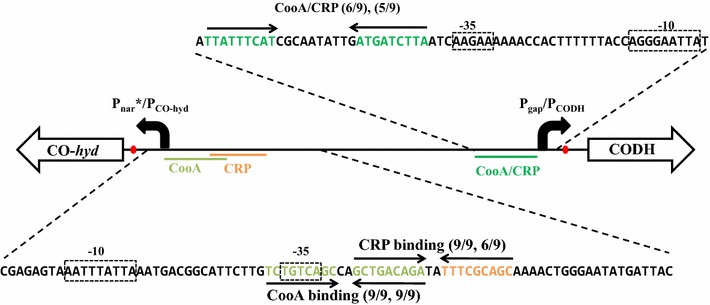
Topology and sequence analysis of CO-regulated promoters in *C. amalonaticus* Y19. The putative CRP- and CooA-binding sites are indicated with *arrows*, and the promoter elements (−10/−35) are shown in *boxes*. The *numbers in parentheses* after CooA and/or CRP indicate matches between each inverted repeat and the consensus sequence motifs (CooA or CRP). The CooA-binding inverted repeat towards the CO-*hyd* operon is a perfectly symmetrical (9/9, 9/9), 9-bp match with that of the CooA/CRP operator reported in *Rhodospirillum rubrum* and other carboxydotrophs (TGTC(A/G)N6(C/T)GACA)

Transcription of the CODH and CO-Hyd operons required both the presence of CO (as the inducer) and the absence of glucose. Positive control by CO is mediated through the transcriptional activator CooA [8, 13], and expression of genes under CCR is often induced by cAMP receptor protein (CRP) [14]. Each of these transcription activator proteins (CooA and CRP) are known to bind as a dimer to regulatory DNA sequences upstream of the −35 region and to interact with the C-terminal domain of the α-subunit (α-CTD) of RNA polymerase [15]. To understand this dual transcriptional control, the intergenic region between the CODH and CO-*hyd* operons was analyzed (Fig. 2). It was confirmed that the two operons were transcribed inversely from the promoters present in the intergenic regions containing the overlapped binding motifs that possibly recruit the two transcription activator proteins, CooA and CRP. Conserved palindromic or semi-palindromic binding motifs for CooA (‘TCTGTCAGC-(N2)-GCTGACAGA’; [16]) and CRP (‘GCTGACAGA-(N2)-TTTCGCAGC’, ‘TTATTTCAT-(N10)-ATGATCTTA’; [17]) were identified for both the CODH and CO-*hyd* operons (Fig. 2). The putative CooA-binding sites for regulating the transcription of the CO-hyd and CODH operons are composed of two 9 bp conserved fragments 2 and 10 bp apart, respectively. The inverted repeat towards CO-*hyd* was highly symmetrical, a conserved perfect 9-bp match with that of the CooA operator reported for *Rhodospirillum rubrum* [8]. However, the CooA/CRP inverted repeat towards CODH was relatively less conserved, 5 and 6 bp among nine base-pairs matched with conserved CooA/CRP sequence motifs. Similarly, the CRP-binding site towards the CO-*hyd* operon was also weakly conserved, with 6 bp sequence conservation with the conserved 9 bp CRP-binding motif (Fig. 2). Although additional biochemical studies are required, this sequence analysis provides additional evidence for the presence of the dual regulation by both CO and glucose on the CODH and CO-*hyd* operons. Interestingly, the predicted binding motifs for CooA and CRP were partially (for CO-Hyd) or completely (for CODH) overlapped with each other. This arrangement of regulatory regions does not allow simultaneous binding of CooA and CRP, due to steric hindrance. On the contrary, the experimental results suggest the simultaneous involvement (binding) of both transcriptional activator proteins. This conflict can be reconciled if one transcriptional activator protein, most likely CooA in this case, has the ability to make a complex with both CO and cAMP and binds to a single regulatory site of each operon. However, thus far, no such transcriptional activator protein, which becomes activated and acquires DNA binding affinity when two effector molecules (in this case, CO and cAMP) are complexed, has been reported. Further molecular and biochemical studies are required.

### Selection of native constitutive promoters

**Fig. 3:**
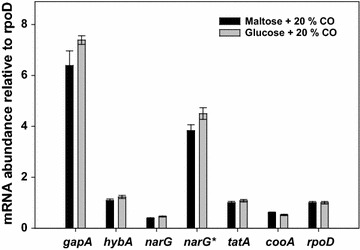
Comparison of promoter strengths of various constitutively expressed genes in *C. amalonaticus* Y19. The relative mRNA abundance of the mutated version of narG* was estimated based on the mRNA abundance of narG in *E. coli. C. amalonaticus* Y19 was grown on glucose or maltose and in the presence of 20% CO

For selection of proper constitutive CODH and CO-*hyd* operon promoters, three strategies/criteria were adopted as follows: (i) the promoters for the two operons should be different from each other so as to obtain a stable construct (to avoid homologous recombination between the promoters); (ii) the strength of the constitutive promoters should be comparable to or stronger than the native inducible promoters for high gene expression; (iii) the promoter of CODH should, according to Fig. 1b, be stronger than that of CO-*hyd*. In order to identify suitable constitutive promoters, transcription of five key enzymes expected to be constitutively expressed at a high level in *C. amalonaticus* Y19 were analyzed by RT-PCR. The enzymes were as follows: glyceraldehyde-3-phosphate dehydrogenase (gapA), uptake hydrogenase (*hybA*), nitrate reductase (narG), Sec-independent protein translocase (*tatA*), and carbonmonoxide operon regulator (*cooA*) (Fig. 3). None of these five enzymes exhibited a significant difference in transcription level by carbon source (glucose vs. maltose) or CO (Fig. 3). The gapA gene was transcribed at the highest level among the five, about threefold higher than *hypC* (the first and most highly expressed gene in the CODH operon; see Fig. 1), thus its promoter (P_gap_) was selected for the expression of the CODH operon. The four other genes were less transcribed than gapA or *hypC,* and none of their promoters was considered a proper one for the expression of the CO-*hyd* operon. Recently, Walker et al. reported that, in *E. coli*, the strength of the constitutive nitrate reductase (narG) promoter was greatly improved upon mutation [18]. If this is the case, the mutated narG promoter (designated as P_nar_*) can be a good one for the CO-*hyd* operon (see Additional file 1: Table S1 for promoter sequences). We decided therefore to evaluate this promoter (along with P_gap_) at the protein/translation level (see below).

The P_gap_ and P_nar_* promoters were examined using GFP as a reporter (see Additional file 2: Figure S1). Non-mutated native P_nar_, as a reference, was also tested. After transformation with recombinant plasmids expressing GFP under the control of each promoter (P_gap_, P_nar_* and P_nar_), *C. amalonaticus* Y19 was cultured to 1.6 O.D. and measured for fluorescence. The specific fluorescences were (AU, arbitrary units): 5.7 × 10^4^ AU/OD for P_gap_, 4.3 × 10^4^ AU/OD for P_nar_*, and 5.3 × 10^3^ AU/OD for P_nar_. The strength of P_nar_* was about 75% that of P_gap_, or about eightfold higher than that of P_nar_; thus, P_nar_* was chosen as a suitable promoter for the CO-*hyd* operon. As in the case of *E. coli*, mutation in P_nar_ greatly improved its strength and, subsequently, GFP fluorescence in *C. amalonaticus* Y19.

### Construction of Y19-PR1 and constitutive transcription of CODH and CO-*hyd*

**Fig. 4:**
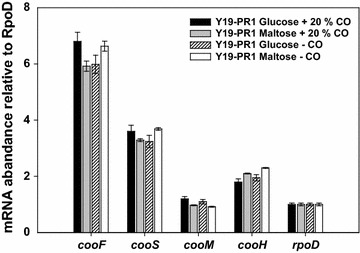
Relative mRNA levels of genes involved in CO-dehydrogenase and CO-dependent hydrogenase in *C. amalonaticus* Y19 and promoter-replaced Y19-PR1 strain. The mRNA levels were compared with those of the reference gene, *rpoD*. *C. amalonaticus* Y19-PR1 recombinant was grown on glucose or maltose and in the presence and absence of 20% CO

Promoter replacement in the chromosome was conducted using a recombinant plasmid pKOV/Δ*P*_*coo*_::*P*_gap–nar_ * containing an integrating fragment (Table 1). It was laborious and took a long time due to the low integration (pop-in) efficiency of the recombinant plasmid to the target site and/or the lack of a proper method to screen the chromosome-integrated recombinants (see “Methods”). The chromosome-integrated, positive recombinants grew very slowly on LB plate under the chloramphenicol selection pressure (25 μM) at 42 °C (the temperature needed for washout of un-integrated plasmids) and were not discriminated from the un-integrated, false-positive ones. After testing >10,000 colonies (by colony PCR) arbitrarily chosen from the chloramphenicol LB plates, only three desired, chromosome-integrated mutants were obtained. Afterwards, the second-round homologous recombination (pop-out) for the excision of the native promoter and plasmid curing were performed by growing the three positive integrants in the presence of sucrose. One final recombinant, designated Y19-PR1, was selected and investigated in detail (for mRNA abundances) (Fig. 4). As expected, in Y19-PR1, transcription of the CODH and CO-*hyd* genes was high, regardless of the type of carbon source and/or CO. This means that, by replacing the promoter region, CCR and CO involvement were fully eliminated. Notably, the transcriptions of *cooF* and *cooS* in Y19-PR1 were ~threefold higher than those in the optimally grown wild-type Y19 (i.e., maltose plus CO). This should be attributed to the fact that the P_gap_ promoter, chosen for CODH, is stronger than the native, CO-inducible promoter. However, the transcription levels of the CO-*hyd* genes in the new construct were two- to threefold lower than those of CODH and, furthermore, not much different from the ones in the optimally grown wild-type Y19. This is somewhat surprising considering that, according to the GFP experiment described above, the strength of P_nar_* was close to that of P_gap_. The genotype of the final recombinant strain (Y19-PR1) is shown in Additional file 3: Table S3.

### Characterization of promoter-engineered strain Y19-PR1

**Fig. 5:**
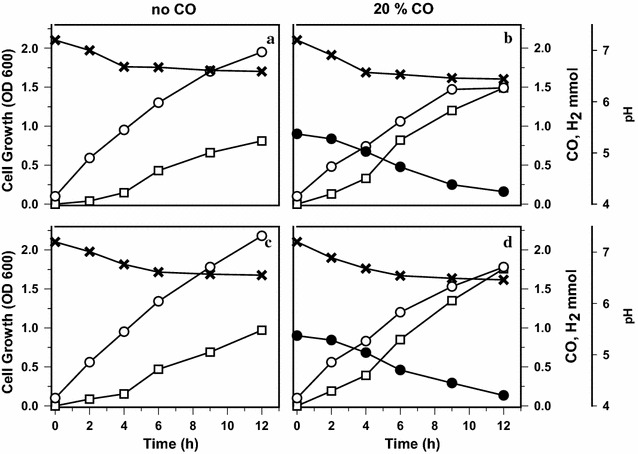
Time-course profiles of cell growth and H_2_ production by wild-type Y19 and Y19-PR1 grown on maltose as carbon source. **a**, **b** Represent wild-type *C. amalonaticus* Y19 while **c** and **d** represent Y19-PR1. Each strain was grown in the absence (**a**, **c**) and presence (**b**, **d**) of CO. *Symbols* cell growth (*open circle*), CO consumption (*closed circle*), H_2_ production (*open square*) and pH (*cross*)

**Fig. 6:**
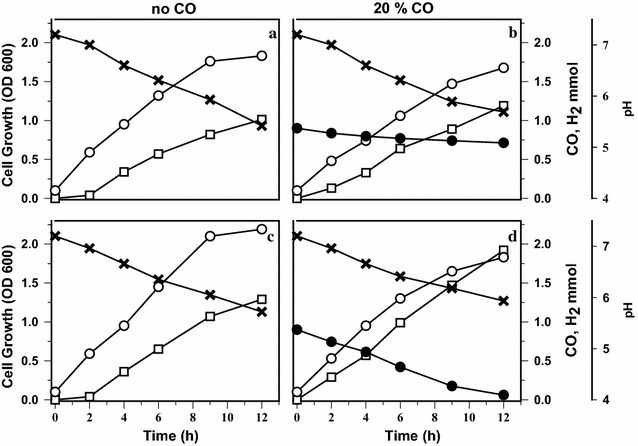
Time-course profiles of cell growth and H_2_ production by wild-type Y19 and Y19-PR1 grown on glucose as carbon source. **a**, **b** Represent wild-type *C. amalonaticus* Y19 while **c**, **d** represent Y19-PR1. Each strain was grown in the absence (**a**, **c**) and presence (**b**, **d**) of CO. *Symbols* cell growth (*open circle*), CO consumption (*closed circle*), H_2_ production (*open squares*) and pH (*cross*)

The cell growth and H_2_ production of the wild-type and promoter-engineered Y19-PR1 strains were examined (Figs. 5, 6). With maltose as the carbon source (Fig. 5), the final cell densities in the presence of CO were 0.53 g L^−1^ for the wild-type and 0.61 g L^−1^ for Y19-PR1. On the other hand, in the absence of CO, they increased to 0.68 g L^−1^ for the wild-type and to 0.76 g L^−1^ for Y19-PR1, respectively. The reason for the growth difference between the two strains, regardless of CO, is not clear. Lee et al. also have reported that replacement of CO-responsive promoters with constitutive promoters significantly improved cell growth in thermophilic archaea *Thermococcus onnurineus* NA1 [19]. The presence of CO reduced the cell growth rate and final cell density in both strains, indicating that CO is toxic. Hydrogen can be generated from CO or formate (by formate hydrogen lyase, FHL) which is derived from maltose metabolism. Without CO (Fig. 5a, c), the same H_2_-production profiles resulted, indicating that the metabolic throughput from maltose to H_2_ is the same in the two strains. On the other hand, when cultured in the presence of CO, both strains showed improved H_2_ production due to the additional production of H_2_ from CO oxidation. The CO assimilation and H_2_ evolution in Y19-PR1 were almost the same as or even better than those in wild-type Y19, indicating that the constitutive promoters were functioning properly (Fig. 5).

With glucose as the carbon source (Fig. 6), Y19-PR1 also showed better cell growth and H_2_ production than did wild-type Y19. The negative effect of CO on cell growth was similar to that when cells were grown on maltose (Fig. 5). In wild-type Y19 growing on glucose, CO was marginally utilized and H_2_ production was not increased by CO (Fig. 6a, b). By contrast, in Y19-PR1, CO was actively metabolized, and more H_2_ was accumulated when CO was added. This confirmed that the expression of the CODH and CO-*hyd* operons in Y19-PR1 is free from CCR. The growth advantage of Y19-PR1 over wild-type Y19 can be well appreciated by comparing Figs. 5b and 6c. Under these different but WGSR-activating growth conditions, the recombinant Y19-PR1 showed, compared with the wild type, an ~30% higher specific cell growth rate and >40%-improved cell density after 9 h cultivation.

Next, the CO-linked H_2_-production activity was measured with whole-cells and crude-cell extracts (Table 2). When grown on maltose plus CO, wild-type Y19 showed whole-cell CO-dependent H_2_ activity of 22.1 mmol g^−1^ h^−1^; when CO was omitted, no activity was observed. The recombinant Y19-PR1, however, showed a comparable CO-linked H_2_-production activity regardless of CO. The activities of CODH (CooS) and hydrogenases (CO-Hyd and FHL) were also measured with broken-cell extracts. The CODH activity of Y19-PR1 (±CO) was nearly 1.5 times higher (49.3 µmol min^−1^ mg^−1^) than that of the wild type (+CO and with maltose; 32.1 µmol min^−1^ mg^−1^). However, the MV-dependent hydrogenase activities (which are the sum of the CO-Hyd and FHL activities) were almost the same for the wild type (2.5 µmol min^−1^ mg^−1^) and Y19-PR1 (2.3 µmol min^−1^ mg^−1^), when grown with CO. We believe that the two strains (for wild type, grown on maltose with CO) have similar CO-Hyd activity, although it could not be determined separately from that of FHL in cell lysates. Both the similar activities in the whole-cell FHL (Table 2) and the broken-cell evolving hydrogenases in the two strains support our hypothesis. Collectively, these results demonstrate the advantageous characteristics of Y19-PR1; whole-cell CO-dependent H_2_ and broken-cell CODH yields were high and not affected by the carbon source or CO.

**Table 2:** Specific activities in whole-cells and broken-cell extracts

Cell^a^	Whole-cells CO-dependent H_2_ production (mmol g^−1^ h^−1^)	Formate-dependent H_2_ production (mmol g^−1^ h^−1^)	Crude-cell lysates CODH (μmol min^−1^ mg^−1^)	Evolving hydrogenase (μmol min^−1^ mg^−1^)	Uptake hydrogenase (μmol min^−1^ mg^−1^)
Cultivated on maltose					
Y19 WT (no CO)	ND	16.2 ± 0.7	ND	1.6 ± 0.1	9.8 ± 0.5
Y19 WT (20% CO)	22.1 ± 1.1	12.0 ± 0.6	32.1 ± 6.1	2.5 ± 0.2	7.9 ± 0.4
Y19-PR1 (no CO)	23.8 ± 1.2	15.2 ± 0.7	49.3 ± 6.7	2.8 ± 0.2	8.9 ± 0.5
Y19-PR1 (20% CO)	22.9 ± 1.2	10.7 ± 0.5	45.6 ± 6.7	2.3 ± 0.1	8.2 ± 0.4
Cultivated on glucose					
Y19 WT (no CO)	ND	18.2 ± 0.8	ND	1.9 ± 0.2	12.4 ± 0.7
Y19 WT (20% CO)	3.7 ± 0.2	14.0 ± 0.7	2.1 ± 0.1	3.2 ± 0.1	9.1 ± 0.4
Y19-PR1 (no CO)	19.8 ± 0.9	17.2 ± 0.9	36.3 ± 1.8	3.6 ± 0.2	11.9 ± 0.6
Y19-PR1 (20% CO)	20.7 ± 1.1	15.3 ± 0.7	41.9 ± 2.7	3.3 ± 0.2	8.9 ± 0.4

*CODH* CO dehydrogenase, *CO*-*hyd* CO-dependent hydrogenase, *ND* not detected

^a^Cells were grown in either presence (20%, v/v) or absence of CO in the head space

### Efforts to improve CO-dependent H_2_ production

Despite successful construction of Y19-PR1 strain, its CO-linked H_2_-production activity was not improved relative to that of its wild-type counterpart. According to RT-PCR analyses (Fig. 4) and activity measurement (Table 2), the expression and enzymatic activity of CO-Hyd were not improved. We speculated that CO-Hyd activity is rate limiting, and thus we decided to improve its expression in Y19-PR1.

Two additional recombinants were constructed. First, the five genes of the CO-*hyd* operon, *cooKLXUH*, were homologously overexpressed, episomally, using the low-copy plasmid pDK7-p15A (under the control of P_nar_* promoter *pHyd*-CO), while the *cooM* gene was moderately up-regulated from the chromosome by engineering of 5′UTR (the untranslated region). The CooM is a large (3789 bp) and membrane-embedded protein with 36 trans-membrane helices (as predicted by the TMPred tool [20]); as such, there has been concern that its overexpression is potentially fatal to cell viability. Various synthetic 5′UTR (including RBS) were designed for CooM using the UTR Library Designer (Additional file 4: Table S3) [21, 22]; two of them, which were expected to provide ~threefold (designated as ‘PR2′) and ~sevenfold (designated as ‘PR3’) higher translations relative to those of PR1 (where the P_nar_* promoter with native RBS was employed), respectively, were chosen for further studies. When analyzed by GFP, the new RBS showed proper strength: 8.7 × 10^4^ AU with PR2 and 1.5 × 10^5^ AU with PR3, respectively. This corresponds to ~2.1- and ~4.0-fold higher expressions, respectively, relative to that of PR1. The new promoters with improved 5′UTR were integrated into the chromosome of Y19-PR1 to replace the P_nar_* promoter using the pKOV system, and two new host strains, Y19-PR2 and Y19-PR3, were developed. Then, to these two new hosts and the original Y19-PR1 strain, the recombinant plasmid containing *cooKLXUH* genes (pHyd-CO) was introduced. Expression of the CO-*hyd* subunits was analyzed in the new recombinant strains on SDS-PAGE after growing them on both maltose and glucose (Additional file 5: Figure S2). In the Y19-PR2/pHyd-CO and Y19-PR3/pHyd-CO strains, CooM (136 kDa), CooK (33.9 kDa) and CooH (40.2 kDa) were detectable in insoluble fractions (due to their membranous nature), whereas neither CooX (22.1 kDa) nor CooU (19.4 kDa) nor CooL (15.5 kDa) was detected in either soluble or insoluble fractions. In the Y19-PR1/pHyd-CO strain, CooK (33.9 kDa) and CooH (40.2 kDa), but not CooM (136 kDa), were detectable in insoluble fractions. In wild-type Y19 without pHyd-CO, no protein band corresponding to any of the CO-*hyd* genes was evident.

The three recombinant Y19 were cultured and their CO-dependent H_2_ production activity were measured (Additional file 6: Table S4). New hosts Y19-PR2 and Y19-PR3 and their recombinants containing pHyd-CO showed defect in cell growth. The hosts Y19-PR2 and Y19-PR3 overexpressing the membrane-embedded protein CooM (Y19-PR2 and Y19-PR3) showed up to 23% reduced cell growth relative to the Y19-PR1 host (Additional file 4: Table S3). When pHyd-CO was introduced, Y19-PR2/pHyd-CO and Y19-PR3/pHyd-CO, even under the best conditions (on glucose without CO), grew to only 1.04 and 0.81 OD_600_, respectively, by 12 h (vs. 1.7 OD_600_ for Y19-PR1). Furthermore, the whole-cell CO-dependent H_2_-production activities of Y19-PR2/pHyd-CO and Y19-PR3/pHyd-CO were greatly reduced to 16.5 and 15.3 mmol H_2_ g^−1^ cell h^−1^, respectively, which levels were 28 and 33% lower, respectively, than that of Y19-PR1. On the other hand, although Y19-PR1/pHyd-CO did not show any problems in its cell growth characteristics (relative to those of Y19 WT), its CO-dependent H_2_ production capabilities were not enhanced. This indicates that improvement of CO-Hyd activity is highly challenging, and cannot be achieved simply by overexpression of the CO-*hyd* operon.

In another effort to improve CO-Hyd activity, the increase of the free membrane space enabling accommodation of additional membrane proteins such as CooM was attempted (Additional file 7: Text S1, Additional file 8: Figure S3, Additional file 9: Table S5). The inner membrane protein, PS003556, which was considered non-essential for cell growth but was expressed at a high level (according to RT-PCR), was removed from Y19-PR2, and the strain was examined for cell growth and H_2_ production capability both as is and after pHyd-CO introduction. No improvement in cell growth or H_2_ production capability in the host or its recombinant was observed relative to Y19-PR2 or its recombinant. These experiments indicated that improvement of CO-Hyd activity is highly challenging and will require further and more intensive investigation.

## Conclusion

For faster cell growth on glucose and convenient expression of CO-linked H_2_ production activity, the promoter-replaced strain *C. amalonaticus* Y19-PR1 was developed. The engineered Y19-PR1, when grown on glucose, could constitutively express CO-linked H_2_-production activity at a high level without dependence on CO. However, the CO-linked H_2_-production activity in Y19-PR1 was not higher than that in the wild-type counterpart. Some efforts to improve CO-dependent H_2_-production activity, including overexpression of CO-Hyd enzymes and removal of an unnecessary membrane protein, were attempted but were not successful. Further studies to uncover the unique regulatory mechanisms of CODH and CO-Hyd expression at the molecular level and further improve the expression of CO-Hyd are under way.

## Additional files

**Additional file 1: Table S1.** Intergenic regions of various constitutive promoters in *C. amalonaticus* Y19.

**Additional file 2: Figure S1.** Fluorescence responses as controlled by selected native (P_gapA_ and P_narG_) and mutated constitutive promoters (P_narG_*) in *C. amalonaticus* Y19.

**Additional file 3: Table S2.** The gentype of the promoter replaced Y19-PR1 strain.

**Additional file 4: Table S3.** Output generated by ‘UTR designer tool’ to design UTR’s with desired expression with P_nar_* promoter.

**Additional file 5: Figure S2.** Expression of CO-Hyd subunits (CooMKLXUH) on SDS-PAGE in the presence of CO. The insoluble and cell free extracts of the Y19-PR1 (lanes 1, 6 and 10, 15), Y19-PR1 *pHyd*-CO (lanes 2, 7 and 11, 16), Y19-PR2 *pHyd*-CO (lanes 3, 8 and 12, 17) and Y19-PR3 *pHyd*-CO (lanes 4, 9 and 13, 18) respectively, grown anaerobically cultivated on maltose and glucose as carbon source, respectively. Protein marker (lane 5, 14), Fermentas #SM1811. The arrows indicate the expression of CooM, CooK, CooH and CooX at ~136 kDa, 34 kDa, 40 kDa and 22 kDa respectively.

**Additional file 6: Table S4.** Table S4 Cell growth characteristics and CO-dependent H_2_ production capabilities by various recombinant *C. amalonaticus* Y19 strains on glucose in the absence of CO.

**Additional file 7: Text S1.** Text S1 Approach for screening highly expressed membrane protein.

**Additional file 8: Figure S3.** Systematic methodology for the selection of inner-membrane proteins from the proteome of *C. amalonaticus* Y19 that occupies outsized inner membrane space.

**Additional file 9: Table S5.** Relative mRNA expression levels of various inner-membrane proteins in *C. amalonaticus* Y19.

## Abbreviations

CODH: carbon monoxide dehydrogenase
CO-Hyd: carbon monoxide-dependent hydrogenase
FHL: formate hydrogen lyase
