# Supplementary material for: Citrobacter amalonaticus Y19 for constitutive expression of carbon monoxide-dependent hydrogen-production machinery
Source: Biotechnol Biofuels. 2017 Mar 28;10:80. doi: 10.1186/s13068-017-0770-8 (PMC5371261; doi:10.1186/s13068-017-0770-8)
Supplement: Supplementary file 1 — Additional file 1: Table S1. Intergenic regions of various constitutive promoters in C. amalonaticus Y19. [file 13068_2017_770_MOESM1_ESM.docx]

**Additional file 1 : Table S1**

**Table S1.** Intergenic regions of various constitutive promoters in *C. amalonaticus* Y19.

| **Promoter** | **Sequence (5’ – 3’)a** | **Source** |
| --- | --- | --- |
| P_gapA_ | ATGCAAATCACA**TTTTTA**TCTCAGTTGCC**CTTTAAAAT**TCGGGGCGCCGCCCCCATGTGGTTTCAAGCCCAATGGAAGAGTGAGGCGAGTCAGCCGTGCAAAGCTTGTACAGAGGATTGATTTGTCGCAATGATTGACACGATTCCGC**TTGACG**CTGCGTAAGGT**TTTTGTAAT**TTTACAGGCAACCTTTTATTCACTAACAAATAGCTGGTGGAATAT | This study |
| P_hybA_ | TATTATCTTGCTTTAATTAATTACAC**TAGCCG**CCACTCCCTTCG**TTTTATTAT**TCCCGCGCAGTATTATAGGCTACATAACCATAACAAAAGTGTGGTAAATGGCGCAGCGATCGCAGTTACGGATTTGTGATTGTTGCAAAATAAGAGCCAGGTCTTCGTAACGGAATAACTATAAA | This study |
| P_narG_ | CCTTTCTGCCTTGATCGTTATCAA**TTCTCA**CGCCCTTTCAGAG**CGTTACCTT**CGCTCTCAATCAAGCAATGTCGATTTTTCTGAATGTCGATTTATCAGAGAGCCGTCAGGCTCCACAGGAGAGAACCG | This study |
| P_narG*_ | CCTTTCTGCCTTGATCGTTATCAA**TTCTCA**CGCCCTTTCAGAG**CGTTATAAT**CGCTCTCAATCAAGCAATGTCGATTTTTCTGAATGTCGATTTATCAGAGAGCCGTCAGGCTCCACAGGAGAGAACCG | This study |
| P_tatA_ | GCCCGGTTGGTTAATGGCGGGCGGTCTGGTCGCCTGGATTGTCGGCTGGCGCAAAACTCGCTGATTTT**TTCATC**GCTCAAGGCAGGCCGGGTA**ACGTATACT**ACGGCCTTAATAATTCATCATCTATCACAGAGGAACATGT | This study |
| P_cooA_ | AGCAATGATTCGGCTACTGGTATCTCAACTGAAATTCTATCTCGCTGGATTCACGTTGCTGTTTCCTCAGCAGCTTCCTGCCGGCGATGGATCCATTGCGTCTGGGCAGGCAGTTGTCGCCTCTGCCCG**CTGCAA**AGCTCATTGAGCAA**AGCAATGAT**ACTTGTATGTCCTTATCGTGTGTATGCGGAAGTGAATAGCAGAGAGGTCGGCA | This study |
