# Supplementary material for: Citrobacter amalonaticus Y19 for constitutive expression of carbon monoxide-dependent hydrogen-production machinery
Source: Biotechnol Biofuels. 2017 Mar 28;10:80. doi: 10.1186/s13068-017-0770-8 (PMC5371261; doi:10.1186/s13068-017-0770-8)
Supplement: Supplementary file 2 — Additional file 2: Figure S1. Fluorescence responses as controlled by selected native (PgapA and PnarG) and mutated constitutive promoters (PnarG*) in C. amalonaticus Y19. [file 13068_2017_770_MOESM2_ESM.docx]

**Additional file 2 : Fig. S1**

**Fig. S1** Fluorescence responses as controlled by selected native (P*_gapA_* and P*_narG_*) and mutated constitutive promoters (P*_narG*_*) in *C. amalonaticus* Y19.
