# Supplementary material for: Citrobacter amalonaticus Y19 for constitutive expression of carbon monoxide-dependent hydrogen-production machinery
Source: Biotechnol Biofuels. 2017 Mar 28;10:80. doi: 10.1186/s13068-017-0770-8 (PMC5371261; doi:10.1186/s13068-017-0770-8)
Supplement: Supplementary file 3 — Additional file 3: Table S2. The gentype of the promoter replaced Y19-PR1 strain. [file 13068_2017_770_MOESM3_ESM.docx]

**Additional file 3 : Table S2**

**Table S2.** Genotype of mutant *C. amalonaticus* Y19 (Y19-PR1).

| **Promoter** | **Sequence (5’ – 3’)a** | **Source** |
| --- | --- | --- |
| Y19-PR1 | CAGCGTCGCGATTGCGGCAATGGCCCATTCCCCTTGTTCCATTGCGCCATATAAAATCAGAAACTTGCTGAATGAGCCTTTAAAGGGGGACAGTCCCATTACGGAAAACAGACCGAAGCCAAACAGCAATGACGCCAGTGGCCAGCGTGAACCGCTGCCTGCCAGCTTATCCAGCTCACTTGAGCAGTTACGCTTAATAAGATACGCCGCACTGAGGATGGTCAACAGGCGCATAACCACCTGTAACCCCAAATGCATCAACGCGCCGGTTTCACCCGATGCGCTATCAAGACCAAAACCCAACAATACATAGCCCAGCTCTGCCAGCGTGGAAAAAAGCAGTAGCCTGGACAGATTGCGGATATGTAATAACGCCATTACTTCACCGGCTAATAATATTAATGTTCCTGACCATGCGAGTAATGGAATGCCGAACAATGATACTCCCATCGGTTCTCTCCTGTGGAGCCTGACGGCTCTCTGATAAATCGACATTCAGAAAAATCGACATTGCTTGATTGAGAGCGATTATAACGCTCTGAAAGGGCGTGAGAATTGATAACGATCAAGGCAGAAAGGGATGAAAACGCGGGGTAAATTTTGACATACCCCTTTCGGATGATTTGATTTATTTGATTATTGCATTGATTGAAAACACGATAGGTAAAATAATTAATAACTCAGTTGCCCTTTAAAATTCGGGGCGCCGCCCCCATGTGGTTTCAAGCCCAATGGAAGAGTGAGGCGAGTCAGCCGTGCAAAGCTTGTACAGAGGATTGATTTGTCGCAATGATTGACACGATTCCGCTTGACGCTGCGTAAGGTTTTTGTAATTTTACAGGCAACCTTTTATTCACTAACAAATAGCTGGTGGAATATATGAAAAAAAAGGAAAATGTCATGATTTACGCCAATGCGCAGAAATGCCTTGGCTGCCATAGCTGTGAAATGGCCTGCGCGGTCAGTCACGGCGGGGATAACGGGTTGTTTGATGCGGTTCGGGACCAGCTTCCGCTCCATCCACGCATGAAGGTGGTGGCAACGGGCGAGGCCAATATTCCGATGCAGTGCCGCCAGTGTGATAACGCCCCCTGCGCGATGGTCTGCCCCACCGGAGCCTGCCGTCAAACGGACGGACAGGTGTTTATCAATGAGGCCAACTGCGTCGGCTGCAAGCTGTGCGTGATGGTCTGTCCGTTTGGCTCTATCACCGTACGGCGCACCGAGCAGCAAAGCGCCTGGAGCGTGACCAACCAGGGCGTGGCGCAAAAATGCGACCTGTGCGTCTCCTGGCGTAAAGAAA | This study |

* Highlighted in Green and Turquoise are P_nar_* and P_gap_ promoters respectively.
