# Supplementary material for: Citrobacter amalonaticus Y19 for constitutive expression of carbon monoxide-dependent hydrogen-production machinery
Source: Biotechnol Biofuels. 2017 Mar 28;10:80. doi: 10.1186/s13068-017-0770-8 (PMC5371261; doi:10.1186/s13068-017-0770-8)
Supplement: Supplementary file 4 — Additional file 4: Table S3. Output generated by ‘UTR designer tool’ to design UTR’s with desired expression with Pnar* promoter. [file 13068_2017_770_MOESM4_ESM.docx]

**Additional file 4 : Table S3**

**Table S3**. Output generated by ‘UTR designer tool’ to design UTR’s with desired expression with P*_nar_** promoter.

| **Start** | **Spacing** | **5'-UTR Sequence** | **Protein Coding Sequence** | **tAI** | **dG_UTR_** | **Expression Level** |
| --- | --- | --- | --- | --- | --- | --- |
| **25** | **6** | **TGTTCTTGGGAAAGGAGGATCGCGG** | **ATGGGAGTATCATTGTTCGGCATTCCATTACTCGC** | **0.22** | **-9.24** | **1,395,906** |
| **25** | **6** | **TGTTCTCGGGAAAGGAGGATCGCGG** | **ATGGGAGTATCATTGTTCGGCATTCCATTACTCGC** | **0.22** | **-8.94** | **1,180,282** |
| **25** | **6** | **TGTTCTTGGGAAAGGAGGATCCCGG** | **ATGGGAGTATCATTGTTCGGCATTCCATTACTCGC** | **0.22** | **-5.94** | **220,436 (B)** |
| **25** | **6** | **TGTTCTCGGGAAAGGAGGATCCCGG** | **ATGGGAGTATCATTGTTCGGCATTCCATTACTCGC** | **0.22** | **-5.54** | **176,247** |
| **25** | **6** | **TGTTCTCGGGAAAGGAGCATCGCGG** | **ATGGGAGTATCATTGTTCGGCATTCCATTACTCGC** | **0.22** | **-4.49** | **97,966 (A)** |
| **25** | **6** | **TGTTCTTGGGAAAAGAGGATCGCGG** | **ATGGGAGTATCATTGTTCGGCATTCCATTACTCGC** | **0.22** | **-3.64** | **60,899** |
| **25** | **6** | **TGTTCTCGGGAAAGGAGCATCCCGG** | **ATGGGAGTATCATTGTTCGGCATTCCATTACTCGC** | **0.22** | **-3.59** | **59,220** |
| **25** | **6** | **TGTTCTTGGGAAAGGAGCATCGCGG** | **ATGGGAGTATCATTGTTCGGCATTCCATTACTCGC** | **0.22** | **-3.44** | **54,454** |
| **25** | **6** | **TGTTCTCGGGAAAAGAGGATCGCGG** | **ATGGGAGTATCATTGTTCGGCATTCCATTACTCGC** | **0.22** | **-2.84** | **38,930** |
| **25** | **6** | **TGTTCTTGGGAAAGGAGCATCCCGG1** | **ATGGGAGTATCATTGTTCGGCATTCCATTACTCGC** | **0.22** | **-2.54** | **32,917** |
| **25** | **6** | **TGTTCTTGGGAAAAGAGGATCCCGG** | **ATGGGAGTATCATTGTTCGGCATTCCATTACTCGC** | **0.22** | **-0.09** | **8,362** |
| **25** | **6** | **TGTTCTCGGGAAAAGAGGATCCCGG** | **ATGGGAGTATCATTGTTCGGCATTCCATTACTCGC** | **0.22** | **0.26** | **6,875** |
| **25** | **6** | **TGTTCTCGGGAAAAGAGCATCGCGG** | **ATGGGAGTATCATTGTTCGGCATTCCATTACTCGC** | **0.22** | **1.31** | **3,821** |
| **25** | **6** | **TGTTCTCGGGAAAAGAGCATCCCGG** | **ATGGGAGTATCATTGTTCGGCATTCCATTACTCGC** | **0.22** | **2.21** | **2,310** |
| **25** | **6** | **TGTTCTTGGGAAAAGAGCATCGCGG** | **ATGGGAGTATCATTGTTCGGCATTCCATTACTCGC** | **0.22** | **2.51** | **1,953** |
| **25** | **6** | **TGTTCTTGGGAAAAGAGCATCCCGG** | **ATGGGAGTATCATTGTTCGGCATTCCATTACTCGC** | **0.22** | **3.41** | **1,181** |

1. UTR with expression levels ~3-fold higher than the native RBS of ‘*nar*’ gene in *C. amalonaticus* Y19, Y19-PR2.
2. UTR with expression levels ~7-fold higher than the native RBS of ‘*nar*’ gene in *C. amalonaticus* Y19, Y19-PR3.
