# Supplementary material for: Citrobacter amalonaticus Y19 for constitutive expression of carbon monoxide-dependent hydrogen-production machinery
Source: Biotechnol Biofuels. 2017 Mar 28;10:80. doi: 10.1186/s13068-017-0770-8 (PMC5371261; doi:10.1186/s13068-017-0770-8)
Supplement: Supplementary file 5 — Additional file 5: Figure S2. Expression of CO-Hyd subunits (CooMKLXUH) on SDS-PAGE in the presence of CO. The insoluble and cell free extracts of the Y19-PR1 (lanes 1, 6 and 10, 15), Y19-PR1 pHyd-CO (lanes 2, 7 and 11, 16), Y19-PR2 pHyd-CO (lanes 3, 8 and 12, 17) and Y19-PR3 pHyd-CO (lanes 4, 9 and 13, 18) respectively, grown anaerobically cultivated on maltose and glucose as carbon source, respectively. Protein marker (lane 5, 14), Fermentas #SM1811. The arrows indicate the expression of CooM, CooK, CooH and CooX at ~136 kDa, 34 kDa, 40 kDa and 22 kDa respectively. [file 13068_2017_770_MOESM5_ESM.docx]

**Additional file 5: Fig. S2**


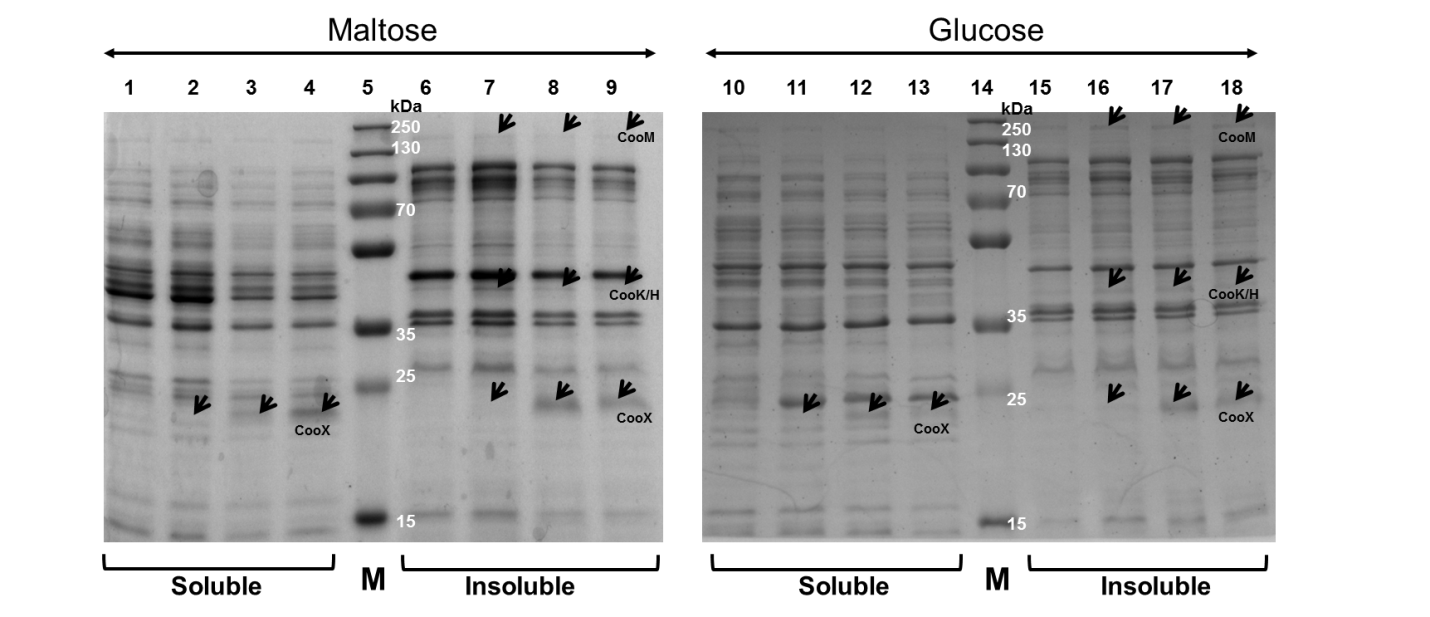


**Fig. S2** Expression of CO-Hyd subunits (CooMKLXUH) on SDS-PAGE in the presence of CO. The insoluble and cell free extracts of the Y19-PR1 (lanes 1, 6 and 10, 15), Y19-PR1 *pHyd*-CO (lanes 2, 7 and 11, 16), Y19-PR2 *pHyd*-CO (lanes 3, 8 and 12, 17) and Y19-PR3 *pHyd*-CO (lanes 4, 9 and 13, 18) respectively, grown anaerobically cultivated on maltose and glucose as carbon source, respectively. Protein marker (lane 5, 14), Fermentas #SM1811. The arrows indicate the expression of CooM, CooK, CooH and CooX at ~136 kDa, 34 kDa, 40 kDa and 22 kDa respectively.
