# Supplementary material for: Citrobacter amalonaticus Y19 for constitutive expression of carbon monoxide-dependent hydrogen-production machinery
Source: Biotechnol Biofuels. 2017 Mar 28;10:80. doi: 10.1186/s13068-017-0770-8 (PMC5371261; doi:10.1186/s13068-017-0770-8)
Supplement: Supplementary file 6 — Additional file 6: Table S4. Table S4 Cell growth characteristics and CO-dependent H2 production capabilities by various recombinant C. amalonaticus Y19 strains on glucose in the absence of CO. [file 13068_2017_770_MOESM6_ESM.docx]

**Additional file 6: Table S4**

**Table S4** Cell growth characteristics and CO-dependent H_2_ production capabilities by various recombinant *C. amalonaticus* Y19 strains on glucose in the absence of CO.

| Strains | Maximum Cell Density (OD_600_) | CO-dependent H_2_ production (mmol g^-1^ h^-1^) |
| --- | --- | --- |
| Y19 WT* | 1.51 | 3.7 ± 0.2 |
| Y19-PR1 | 1.74 | 20.7 ± 1.1 |
| Y19-PR2 | 1.47 | 17.7 ± 2.1 |
| Y19-PR3 | 1.35 | 14.9 ± 2.9 |
| Y19-PR1/pHyd-CO | 1.45 | 18.2 ± 0.8 |
| Y19-PR2/pHyd-CO | 1.04 | 16.5 ± 1.8 |
| Y19-PR3/pHyd-CO | 0.81 | 15.3 ± 2.5 |

* Cultures were grown on glucose in the presence of CO.
