# Supplementary material for: Citrobacter amalonaticus Y19 for constitutive expression of carbon monoxide-dependent hydrogen-production machinery
Source: Biotechnol Biofuels. 2017 Mar 28;10:80. doi: 10.1186/s13068-017-0770-8 (PMC5371261; doi:10.1186/s13068-017-0770-8)
Supplement: Supplementary file 7 — Additional file 7: Text S1. Text S1 Approach for screening highly expressed membrane protein. [file 13068_2017_770_MOESM7_ESM.docx]

**Additional file 7: Text S1.**

**Approach for screening highly expressed membrane protein**

To increase of the free membrane space enabling accommodation of additional membrane proteins, we decided to remove some proteins from the cellular membrane to increase space and, to accommodate more CO-Hyd-complex proteins. CO-Hyd enzyme complex consists of at least 7 proteins, among which two large ones, CooM (MW 136.1 kDa) and CooK (MW 33.9 kDa), are membrane-embedded [23]. To effectively increase the membrane space, the target proteins should be highly expressed and, when deleted, should not affect cell growth and CO-linked H_2_ production. To identify proper target proteins, the entire proteome of *C. amalonaticus* Y19 [23] was screened systematically (Additional file 6: Fig. S3). Briefly, using the TMPred tool, the inner-membrane proteins were screened based on the presence of the signal peptide (which brings proteins into the membrane) and the capability to form trans-helices in the membrane, after which the proteins were examined for their length, number of trans-helices and their location in the chromosome. Finally, the mRNA levels of the selected periplasmic (inner-membrane) proteins were compared with *cooM* (the major subunit gene of the CO-Hyd complex) under both aerobic and anaerobic conditions (Additional file 7: Table S4). Based on the results, ORF PS003556, an inner-membrane protein from the genome of *C. amalonaticus* Y19 was selected as target to be deleted. Deletion of PS003556 from the chromosome did not affect cell growth or H_2_-production activity in Y19-PR1 or its recombinants.
