# Supplementary material for: Citrobacter amalonaticus Y19 for constitutive expression of carbon monoxide-dependent hydrogen-production machinery
Source: Biotechnol Biofuels. 2017 Mar 28;10:80. doi: 10.1186/s13068-017-0770-8 (PMC5371261; doi:10.1186/s13068-017-0770-8)
Supplement: Supplementary file 8 — Additional file 8: Figure S3. Systematic methodology for the selection of inner-membrane proteins from the proteome of C. amalonaticus Y19 that occupies outsized inner membrane space. [file 13068_2017_770_MOESM8_ESM.docx]

**Additional file 8: Fig. S3**

**Fig. S3** Systematic methodology for the selection of inner-membrane proteins from the proteome of *C. amalonaticus* Y19 that occupies outsized inner membrane space.
