# Supplementary material for: Citrobacter amalonaticus Y19 for constitutive expression of carbon monoxide-dependent hydrogen-production machinery
Source: Biotechnol Biofuels. 2017 Mar 28;10:80. doi: 10.1186/s13068-017-0770-8 (PMC5371261; doi:10.1186/s13068-017-0770-8)
Supplement: Supplementary file 9 — Additional file 9: Table S5. Relative mRNA expression levels of various inner-membrane proteins in C. amalonaticus Y19. [file 13068_2017_770_MOESM9_ESM.docx]

**Additional file 9: Table S5**

**Table S5** Relative mRNA expression levels of various inner-membrane proteins in *C. amalonaticus* Y19.

| ***C. amalonaticus* Y19** | **Expression levels** | |
| --- | --- | --- |
| **Gene ID** | **Aerobic** | **Anaerobic** |
| PS00725 | 0.430 | 0.470 |
| PS01037 | 0.370 | 0.420 |
| PS01465 | 0.500 | 0.690 |
| PS04105 | 0.465 | 0.560 |
| PS04361 | 0.940 | 0.540 |
| PS04920 | 0.360 | 0.470 |
| PS00162 | 0.297 | 0.310 |
| PS00532 | 0.864 | 0.677 |
| PS00740 | 0.552 | 0.480 |
| PS00873 | 0.423 | 0.328 |
| PS01047 | 0.627 | 0.563 |
| PS01400 | 0.610 | 0.566 |
| PS01662 | 0.446 | 0.653 |
| PS02170 | 0.390 | 0.410 |
| PS03303 | 0.470 | 0.470 |
| PS03556 | 0.430 | 0.700 |
| PS03659 | 0.390 | 0.540 |
| FHL (*hycC*) | 0.408 | 0.880 |
| CODH (*cooM*) | 0.400 | 0.660 |
